# Early vs Late Initiation of Extracorporeal Membrane Oxygenation: Protocol for a Prospective, Randomized, Multicenter Study

**DOI:** 10.2196/86652

**Published:** 2026-06-17

**Authors:** Alice Bernard, Helene Haeberle, Valbona Mirakaj, Manola Zago, Michael Koeppen, Christina-Katharina Fodi, Peter Martus, Peter Rosenberger

**Affiliations:** 1 Department of Anaesthesiology and Intensive Care Medicine Tübingen University Hospital Tübingen Germany; 2 Center for Clinical Studies Tübingen University Hospital Tübingen Germany; 3 Institute for Clinical Epidemiology und Applied Biostatistics, Eberhard-Karls University Tübingen Germany

**Keywords:** acute respiratory distress syndrome, ARDS, extracorporeal membrane oxygenation, ECMO, inflammation, intensive care, ventilation

## Abstract

**Background:**

Acute respiratory distress syndrome (ARDS) is characterized by severe inflammatory lung injury leading to life-threatening hypoxemia. Standard treatment includes lung-protective mechanical ventilation and adjunctive measures, while veno-venous extracorporeal membrane oxygenation (vvECMO) is used as a rescue therapy in refractory cases. However, the optimal timing for initiation of vvECMO remains uncertain, with official recommendations identifying it as a rescue therapy, while emerging evidence suggests that earlier implementation of vvECMO during the disease course might provide benefits.

**Objective:**

The ELIEO (Early vs Late Initiation of vvECMO) trial aims to determine whether early initiation of vvECMO improves survival and clinical outcomes compared with a conventional strategy in patients with severe ARDS.

**Methods:**

ELIEO is a prospective, randomized, multicenter clinical trial enrolling 508 adult patients with severe ARDS. Participants will be randomized to one of two groups: (1) early vvECMO initiation within 24 hours after admission to the intensive care unit of an extracorporeal membrane oxygenation center or (2) conventional management according to ARDS Network guidelines, with vvECMO used only as rescue therapy. All-cause mortality at day 90 will serve as the primary outcome and will be analyzed using a 2-sided log-rank test within an O’Brien-Fleming group-sequential design. Secondary end points include Sequential Organ Failure Assessment scores, functional status at days 28 and 90, bleeding events, and intensive care unit–related complications, which will be analyzed using appropriate regression and nonparametric methods, with adjusted Cox models for sensitivity analyses.

**Results:**

Patient recruitment started on March 1, 2025, and is ongoing; study completion is expected in August 2028. An interim analysis is planned after the 94th patient has been enrolled. The trial is designed to evaluate whether early initiation of vvECMO reduces 90-day mortality and improves organ function and functional recovery compared with a conventional rescue strategy. Results will be reported after completion of enrollment and follow-up. As of May 2026, a total of 9 patients have been recruited.

**Conclusions:**

The ELIEO trial will provide robust evidence regarding the optimal timing of vvECMO initiation in patients with severe ARDS. The findings may influence clinical decision-making, resource allocation, and organizational strategies for the management of ARDS in specialized intensive care settings.

**Trial Registration:**

ClinicalTrials.gov NCT04208126; https://clinicaltrials.gov/study/NCT04208126

**International Registered Report Identifier (IRRID):**

DERR1-10.2196/86652

## Introduction

Acute respiratory distress syndrome (ARDS) is defined as pulmonary compromise with bilateral pulmonary infiltrates associated with moderate to severe hypoxemia [1]. The public health impact of ARDS is considerable, and it is estimated that up to 50,000 cases of ARDS occur in Germany annually. The estimated mortality ranges from 26% to 51% and depends on the severity of the associated hypoxemia [2]. Patients with ARDS who survive treatment have reduced functional capacity in their everyday life following hospitalization [3,4]. The central pathophysiological changes in patients with ARDS include dysregulated inflammation within the alveolar space, inappropriate accumulation of leukocytes and platelet-neutrophil complexes, uncontrolled activation of coagulation pathways, and altered permeability of the alveolar-capillary barrier [5]. Frequent causes of ARDS are trauma, sepsis, pneumonia, blood transfusion, and aspiration of material into the lungs.

Treatment of ARDS is symptomatic, using mechanical ventilation, prone positioning, and, in extreme cases of hypoxia, the initiation of extracorporeal membrane oxygenation (ECMO) [6]. The current evidence concerning the role of veno-venous ECMO (vvECMO) during respiratory failure is still sparse, and only a few prospective trials have examined the role of vvECMO in treating patients with ARDS. The first randomized multicenter trial that showed a potential benefit of ECMO was the CESAR (conventional ventilatory support vs ECMO for severe adult respiratory failure) trial in 2009. In this trial, patients were randomized to be treated at either a community hospital or an ARDS center during the H1N1 epidemic in Australia and New Zealand [7]. Not all patients in the ARDS center group received ECMO, yet all patients received professional treatment at an expert center. This trial revealed that patients who were treated at an expert center had better outcomes than those who were not treated at such centers. The EOLIA (ECMO to rescue acute lung injury in severe ARDS) trial was the next large trial to investigate the role of ECMO in the treatment of patients with ARDS, but this trial was stopped for futility. Patients who received ECMO tended to have better outcomes than those who received standard treatment, yet 35 out of 120 patients crossed over from the conservative treatment arm [8]. Thus, this trial suggests that the initiation of ECMO might result in improved patient outcomes. However, given the significant number of crossover patients in the EOLIA trial, the question remains whether ECMO therapy for patients with ARDS should begin at an earlier or later stage of treatment. Therefore, we aim to combine the questions raised by both the CESAR and EOLIA trials and evaluate whether early vs late initiation of vvECMO results in improved overall outcomes in patients with ARDS. Within current guidelines [1], the use of ECMO is recommended only as a rescue therapy, which represents a late initiation of vvECMO therapy [9,10]. However, data suggest that early initiation of vvECMO might result in better overall outcomes. A retrospective analysis of a patient cohort with 158 cases of severe ARDS found that a shorter duration of invasive mechanical ventilation prior to vvECMO was associated with lower 28-day and in-hospital mortality. Survivors in this analysis had significantly fewer days of invasive ventilation before ECMO than nonsurvivors, and logistic regression suggested that mortality increased with each additional day of pre-ECMO ventilation, supporting the concept that earlier vvECMO may be beneficial [11,12]. Both publications, though, succumb to the limitations of their retrospective design, and sufficient prospective data are still needed. In a systematic review and meta-analysis, Tan et al [13] focused on severe ARDS caused by the COVID-19 pandemic and hypothesized that late initiation of vvECMO therapy might be an independent risk factor for increased mortality; however, the results are controversial because of the heterogeneity of the studies included, possible selection bias within the individual studies, and the fact that the analysis was specific to COVID-19–associated ARDS.

Evidence suggests that vvECMO can be used with a low risk. In the EOLIA (Early vs Late Initiation of ECMO) trial, the overall complication rate was the same in patients receiving ECMO therapy as in patients in the control group [8]. Patients receiving ECMO therapy presented a higher rate of severe bleeding complications (2% vs 1%), while patients in the control group had a higher incidence of stroke (2% vs 6%). The CESAR trial and the Extracorporeal Life Support Organization (ELSO) registry support these findings [1,14]. Therefore, the increased risk of ECMO therapy mainly arises from a slightly increased rate of minor bleeding (approximately 2%-3%). This risk is balanced against the benefits of lung-protective ventilation, reduction in cytokines, improved overall outcome, and protection of pulmonary and extrapulmonary organ function.

Early use of vvECMO therapy carries a slightly increased risk of mild to moderate bleeding. If ECMO therapy is initiated at a later time point, similar bleeding complications may also occur. A dose-response relationship for ECMO therapy cannot yet be specified, as no study has evaluated this relationship to date.

The ELIEO trial aims to contribute evidence and validated data on the most opportune time point for implementation of vvECMO in the disease course of ARDS: whether vvECMO therapy is more beneficial and justified when initiated earlier (eg, Horovitz index <100) or whether its use should be reserved for rescue therapy only, as suggested by the ELSO guidelines [1] (eg, when the Horovitz index is <80).

## Methods

### Study Design

**Figure 1:**
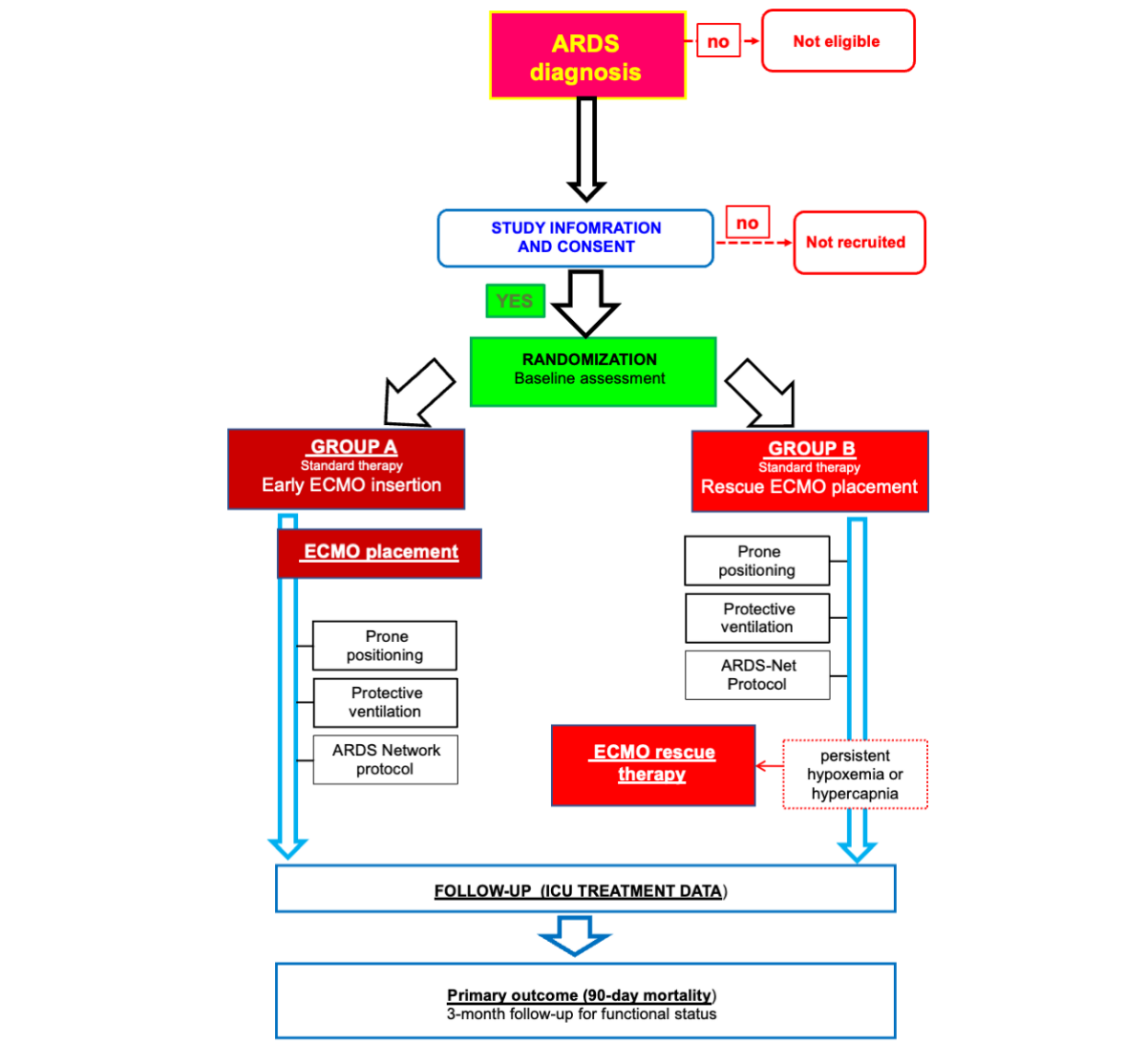
Study flow diagram. After screening and confirmation of eligibility, patients will be enrolled within 24 hours after transfer to an acute respiratory distress syndrome (ARDS) center. During this period, screening, informed consent, and randomization will be initiated. In addition, prone positioning and lung-protective ventilation will be instituted. After randomization, patients will receive either early initiation of extracorporeal membrane oxygenation (ECMO) therapy (group A) or ECMO therapy only as a rescue therapy (group B). Daily recordings will be collected with respect to the development of organ failure and potential adverse events. The follow-up period will continue for up to 90 days to assess patient outcome, quality of life, and pulmonary and secondary organ function. ICU: intensive care unit.

ELIEO is a prospective, randomized, multicenter trial assessing whether early vs late initiation of ECMO could result in improved overall outcomes in patients with ARDS. After providing written informed consent, all patients eligible to participate will be randomized into 2 groups (Figure 1).

The trial is registered at EudraCT (2016-003168-37) and at ClinicalTrials.gov (NCT04208126). Multimedia Appendix 1 gives an overview of the trial registration dataset. The SPIRIT (Standard Protocol Items: Recommendations for Interventional Trials) reporting guidelines were used for the preparation of this protocol [15].

### Ethical Considerations

This study was approved by the local ethics committee of the University of Tübingen, Germany (949/2020BO1), on December 18, 2020. The latest amendment, version 5.0 of the protocol, was approved by the ethics committee on December 20, 2025.

Consent will be obtained from either the patient or a legally authorized representative. Each patient or their legal representative will be informed about the modalities of the clinical study in accordance with the provided patient information. Informed consent will be obtained using a form approved by the ethics committee of the University of Tübingen or the local ethics committee if the patient is treated at a collaborating institution.

The data obtained in the course of the trial will be treated according to the European General Data Protection Regulation (Datenschutz-Grundverordnung) and applicable local data protection regulations. Upon trial inclusion, patients will be pseudonymized using a numerical code (assigned patient number).

To maintain patient privacy, all data capture records, study reports, and communications will identify the patient only by the assigned patient number. The principal investigator determines which persons are authorized to view personal data, and the Patient ID Log is accessible only to authorized study team members. Access rights to personal data (including pseudonymized data) will be restricted to prevent unauthorized access to the data, both electronically and physically. Electronic systems and files are access-regulated and may be password-protected. Documents and files are kept in lockable rooms and, if necessary, in cupboards with access control.

### Trial Sites

All participating trial sites must be able to deliver state-of-the-art care for patients with ARDS. This includes lung-protective ventilation strategies, prone positioning, and bronchoscopy. Participating investigators must have experience treating critically ill patients and patients with ARDS. Standard care for patients with ARDS, with special regard to lung-protective ventilation, is essential. All centers must have experience with ECMO.

### Population

The target population for this clinical trial is critically ill adult patients with severe ARDS. Patients will be randomized into 2 treatment groups:

#### Group A: Early ECMO Initiation

ECMO therapy will be initiated within 24 hours after admission to the intensive care unit (ICU) of the ECMO center or after the inclusion criteria are met. Standard treatment according to ARDS Network guidelines will be provided before and after ECMO therapy is initiated.

#### Group B: Late ECMO Initiation

Standard treatment according to ARDS Network guidelines will be provided, with ECMO therapy used only as rescue therapy. One of the following criteria must be met to permit the use of ECMO therapy as a rescue therapy:

- Inability to administer lung-protective ventilation
- Persistent or progressive hypoxemia (partial pressure of arterial oxygen [PaO_2_]–fraction of inspired oxygen [FiO_2_] <60 mm Hg) or progressive acidosis (pH <7.2) for 6 hours despite corrective measures
- Arterial oxygenation saturation <80% for >6 hours despite mandatory recruitment maneuvers, inhalation of nitric oxide or prostacyclin, and, if technically possible, a trial of prone positioning, without irreversible multiple organ failure

### Inclusion Criteria

Patients diagnosed with ARDS according to the Berlin definition [16], starting from the severe stage with acute onset, who meet the eligibility criteria (Textbox 1) will be included in the trial.

**Eligibility criteria.**

**Inclusion criteria**

- PaO_2_-FiO_2_ ≤100 mm Hg
- One of the following 3 criteria:
  PaO_2_-FiO_2_ ≤100 mm Hg with FiO_2_ >80% for >3 hours despite optimal use of adjunctive therapies
  PaO2-FiO_2_ <80 mm Hg with FiO_2_ ≥80% for >3 hours despite optimization of mechanical ventilation
  pH <7.25 (partial pressure of arterial carbon dioxide ≥50 mm Hg) for >3 hours despite optimal ventilation with a respiratory rate of up to 35 breaths/minute
- Bilateral opacities on frontal chest radiograph within ≤7 days of onset
- Requirement for positive pressure ventilation via an endotracheal tube or noninvasive ventilation
- No clinical signs of left atrial hypertension detected via echocardiography or, if measured, a pulmonary arterial wedge pressure ≤18 mm Hg
- ≤96 hours since the onset of acute respiratory distress syndrome
- ≤7 days since the initiation of mechanical ventilation

**Exclusion criteria**

- Aged <18 years
- >7 days since the initiation of mechanical ventilation
- >96 hours since meeting the inclusion criteria
- Noncommitment from the patient, surrogate, or physician to full intensive care support
- A positive pregnancy test at the time of screening
- Cardiac failure requiring veno-arterial extracorporeal membrane oxygenation
- Chronic respiratory insufficiency treated with oxygen therapy

### Randomization

After the inclusion and exclusion criteria are assessed and informed consent is obtained, the investigator will register patients in the secuTrial database (electronic case report forms [eCRFs]). The study arm of this open-label study will then be transmitted without any delay through computer-generated randomization. Thus, therapy can start immediately. Randomization will be stratified by study center.

### Intervention Plan

#### Overview

This study will consist of the following consecutive phases: study entry, treatment, and follow-up. The time points and trial procedures are listed in Table 1, and the flow of patients within the trial protocol is shown in Figure 1. All patients included in this trial will receive standard treatment according to ARDS Network guidelines, with special consideration of lung-protective ventilation strategies.

**Table 1:** Table of events.

Test Performed	Baseline	Daily during ICU^a^ stay	Days 7, 14, and 28, and at ICU discharge	Day 90
Hematology and coagulation laboratory parameters^b^	✓	✓	✓^c^	
Inflammatory markers^b^	✓	✓	✓^c^	
Renal function parameters^b^	✓	✓	✓^c^	
Ventilation parameters and pressures	✓	✓	✓	
Positioning (prone or supine)	✓	✓	✓	
Partial pressure of arterial oxygen–fraction of inspired oxygen ratio	✓	✓	✓	
Hemodynamic parameters	✓	✓	✓	
Vasopressor and inotropic therapy	✓	✓	✓	
Fluid balance and blood products	✓	✓	✓	
Anticoagulation drugs	✓	✓	✓	
Samples for cytokine array	✓		✓	
Serum samples for protein analysis	✓		✓	
Bronchoalveolar lavage	✓		✓	
Infection status	✓	✓	✓	✓
Anti-infective therapy	✓	✓	✓	✓
ICU length of stay	✓	✓	✓	
Hospital length of stay	✓	✓	✓	✓
Discharge location			✓	✓
Date of death	✓	✓	✓	✓
Cause of death	✓	✓	✓	✓
Richmond Agitation-Sedation Scale score	✓	✓	✓^c^	
Sequential Organ Failure Assessment score	✓	✓	✓^c^	
Barthel Index				✓

^a^ICU: intensive care unit.

^b^Hemoglobin, red blood cells, platelet count, white blood cells, NT-proBNP, hematocrit, procalcitonin, interleukin-6, D-dimers, International Normalized Ratio (INR), factor XIII, factor Xa inhibitor (low molecular weight heparin [LMWH]), urea, creatinine clearance, aspartate transaminase, alanine transaminase, choline esterase, albumin, creatinine kinase, and pregnancy test.

^c^Not required at ICU discharge.

#### Study Entry

Patients with ARDS as defined by the Berlin definition (as depicted in Table 2 and as reported by Ferguson et al [16]) will be screened for eligibility after providing written informed consent.

**Table 2:** Definition of acute respiratory distress syndrome.

Characteristics	Mild	Moderate	Severe
Timing	Within 1 week of a known clinical insult or new or worsening symptoms	Within 1 week of a known clinical insult or new or worsening symptoms	Within 1 week of a known clinical insult or new or worsening symptoms
Oxygenation	PaO_2_^a^-FiO_2_^b^ 200 to ≤300 mm Hg with PEEP^c^ or CPAP^d^ ≥5 cm H_2_O	PaO_2_-FiO_2_ 100 to ≤200 with PEEP or CPAP≥5 cm H_2_O	PaO_2_-FiO_2_ ≤100 mm Hg with PEEP or CPAP ≥5 cm H_2_O
Chest imaging	Bilateral opacities not fully explained by effusions, lobar or lung collapse, or nodules	Bilateral opacities not fully explained by effusions, lobar or lung collapse, or nodules	Bilateral opacities not fully explained by effusions, lobar or lung collapse, or nodules
Origin of edema	Respiratory failure not fully explained by cardiac failure or fluid overload; objective assessment (eg, echocardiography) is required to exclude hydrostatic edema if no risk factors are present	Respiratory failure not fully explained by cardiac failure or fluid overload; objective assessment (eg, echocardiography) is required to exclude hydrostatic edema if no risk factors are present	Respiratory failure not fully explained by cardiac failure or fluid overload; objective assessment (eg, echocardiography) is required to exclude hydrostatic edema if no risk factors are present

^a^PaO_2_: partial pressure of arterial oxygen.

^b^FiO_2_: fraction of inspired oxygen.

^c^PEEP: positive end-expiratory pressure.

^d^CPAP: continuous positive airway pressure.

#### Treatment Phase

Patients assigned to the treatment group will receive vvECMO therapy within 24 hours of arrival at a vvECMO center. The control group will receive vvECMO therapy only as a rescue therapy after conservative therapy has failed (Figure 1). Blood samples will be drawn at admission to test for potential biomarkers to better assess the associations between coagulation, inflammation, and vvECMO treatment. Key cointerventions and mechanical ventilation will be standardized.

#### Follow-Up

Hospital survivors will undergo a brief follow-up phone survey 3 months after enrollment to assess functional status using the Barthel Index [17], frailty using the Vulnerability Elders Survey-13 [18], and health-related quality of life using a standardized questionnaire. Patients will be visited daily until day 28 or until discharge from the ICU. After discharge, patients will next be visited on day 90. Patients remaining in the ICU will continue to receive daily visits until day 90, during which time data collection according to the study procedure will also continue. Each visit will include a clinical examination, collection of a blood sample, assessment of functional capacity using the Barthel Index [17], and assessment of the severity of illness using the Sequential Organ Failure Assessment (SOFA) score. All data will be recorded on eCRFs, which will be used as a visit diary.

### Strategies to Improve Adherence to Interventions

A comprehensive quality control process may be conducted by the coordinating investigator in the form of audits. These audits may include checking the whole course of the study, documentation, statistical analyses, and investigators. The competent regulatory authorities may also conduct audits or inspections.

By participating in the study, the investigator agrees to support the activities of the auditor or inspector, provide them with direct access to source documents, and give them the opportunity to inspect the laboratory facilities, storage of all samples, and related materials.

### Relevant Concomitant Care Permitted or Prohibited During the Trial

Patients will receive standard-of-care treatment within the ICU. This includes all necessary treatments related to fluid management, anti-infective therapy, and blood product administration. There will be no difference between groups regarding this aspect of care. All concomitant medications and treatments are permitted during the trial or prior to enrollment in the clinical trial.

### Outcome Measurements and Study Objectives

The primary objective is overall survival during the 90-day follow-up period (90-day all-cause mortality).

The secondary objectives and end points are provided in Textbox 2.

**Secondary objectives and end points.**

- 28-day all-cause mortality
- Sequential Organ Failure Assessment scores on days 1 to 7, 14, 28, and 90
- Duration of mechanical ventilation support
- Intensive care unit (ICU) length of stay
- Ventilator-associated pneumonia
- Cerebral hemorrhage
- Pulmonary hemorrhage
- Gastrointestinal hemorrhage
- Pulmonary embolism
- Decubitus ulcers
- Delirium
- ICU-acquired weakness
- Discharge location (home, skilled nursing facility, or rehabilitation center)
- Thrombosis

### Data Collection and Management

#### Case Report Form

The trial case report form is the primary data collection instrument for the trial. For this project, eCRFs will be used. Entered data will be subjected to plausibility checks implemented directly in the case report form, as well as monitoring and medical review. The trial master file, the eCRFs, and the other materials supplied for the conduct of the study will be retained by the sponsor or contract research organization according to applicable regulations and laws. The investigator(s) will archive all trial data (source data and Investigator Site File, including the participant ID list and relevant correspondence) according to the International Council for Harmonisation Consolidated Guideline for Good Clinical Practice and local laws and regulations.

#### Sample Size

We assume 90-day survival rates of 63% in the ECMO group and 50% in the control group [14]. Conservatively, we calculate survival rates for 60 days, even though we will have a follow-up period of 90 days because of the assumption that survival curves approximately reach a plateau after 60 days. We require 220 evaluable patients per group, with 187 events in total, to achieve a power of 80%. Assuming a dropout rate of 10%, which seems to be justified due to the relatively short follow-up interval, we need 245 patients per group. Furthermore, we will use a group-sequential design with 1 interim analysis according to O’Brien-Fleming (refer to the Statistical Analyses section). Therefore, we increased the sample size by a factor of 1.008, which leads to 247 patients per group. Increasing the sample size for 16 study centers leads to a total of 508 patients.

To ensure sufficient recruitment, established or expert centers for ECMO treatment will be included as trial sites. In case of insufficient recruitment, additional centers will be included in the trial to recruit patients. We expect a seasonal peak in available patients during fall, winter, and early spring.

### Statistical Analyses

The primary outcome, overall survival, will be analyzed using a 2-sided log-rank test with an overall type 1 error of 0.05 in the intention-to-treat population. An O’Brien-Fleming 2-group design with *P*=.005 and *P*=.048 will be used, and an interim analysis will be performed once 94 events have been observed. The secondary outcomes will be analyzed using chi-square tests (28-day all-cause mortality; secondary end points 5, 6, 7, 8, 9, 10, 11, and 12; and discharge location), mixed models (SOFA score), and Mann-Whitney tests (duration of mechanical ventilation support and ICU length of stay). Further safety analyses will be performed descriptively. Imputation is not planned because, with the exception of long-term survival, we will analyze only routinely documented ICU data.

### Methods for Additional Analyses: Subgroup Analyses and Adjusted Analyses

Secondary analysis of the primary outcome will be adjusted for study center, PaO_2_-FiO_2_ (continuously scaled), baseline SOFA score, sex, and age. No further subgroup analyses are planned. An exploratory prediction model will be built. These analyses will be performed using the Cox proportional hazard model. Additionally, time to ECMO initiation will be analyzed in the late ECMO group by calculating cumulative incidences using the Nelson-Aalen estimator and considering death as a competing risk. Analysis will be performed using SPSS (version 31; IBM Corp) for Windows and the latest version of R (version 4.6.0; R Foundation for Statistical Computing).

## Results

### Patient and Trial Status

The ELIEO trial is an ongoing prospective, randomized, multicenter study evaluating early vs late initiation of vvECMO in patients with severe ARDS. Patient recruitment started on March 1, 2025. Enrollment of the last patient is expected in the first quarter of 2028. The approximate completion of recruitment (last patient out) is expected by the second quarter of 2028. Completion of the statistical analysis and preparation of the trial report are intended for the second quarter of 2029. In total, 508 patients will be recruited. An interim analysis will be performed as described later. This protocol version is version 4, dated March 10, 2025. As of May 2026, a total of 9 patients have been enrolled.

### Data and Safety Monitoring Board and Interim Analysis

An independent Data and Safety Monitoring Board (DSMB) will be assembled. The DSMB will be composed of independent experts in the field of ARDS and an independent biometrician, who will assess progress, safety data, and critical efficacy end points. The mission of the DSMB will be to ensure the ethical conduct of the trial and to protect the safety interests of patients in this trial.

After the 50th enrolled patient completes the day 28 visit, a DSMB meeting will be scheduled. Additional meetings may be scheduled annually or whenever deemed necessary by the members. The DSMB will be informed of the outcome of the interim analysis after 94 events.

The trial may be prematurely terminated if, in the opinion of the sponsor, there is sufficient reasonable cause or based on a recommendation of the DSMB. Written notification documenting the reason for study termination will be provided to the investigators.

In the following situations, premature termination of the trial has to be considered: substantial changes in risk-benefit considerations, new insights from other trials, and insufficient recruitment rates.

The DSMB will monitor study conduct and safety aspects of the trial on a regular basis and will give recommendations to the sponsor regarding whether to stop the trial or to modify the trial protocol. The sponsor will then decide on the actions to be taken. The trial may be suspended or prematurely terminated by decision of the competent authority.

The sponsor will inform investigators of all relevant safety issues and corresponding decisions of the DSMB. If new information becomes known that differs from the scientific information given to the investigator, all investigators will be informed of this by the sponsor.

### Biometric Report

The biometric report will be delivered according to the standard operating procedure BI07 of the statistical center (Institute for Clinical Epidemiology and Applied Biostatistics). In summary, the report will contain sections on statistical methodology; preprocessing of data; and descriptive, exploratory, and confirmatory analyses. This report will be reviewed by the principal investigator before the final version is presented.

### Publication of the Results

The results obtained in this trial will be published in an international journal and may be presented at international scientific meetings. Authorship of any publications will be determined based on contributions to the design, conduct, interpretation, and reporting of the ELIEO trial.

## Discussion

The ELIEO trial is a prospective, randomized, multicenter trial evaluating the timing of vvECMO therapy for the treatment of patients with severe ARDS. The effect of the therapy will be assessed by dividing patients into 2 groups: the experimental group will receive vvECMO therapy as early as possible, whereas the control group will receive ECMO therapy only as a last resort, as outlined in the current ARDS Network guidelines.

Prior studies have shown that vvECMO likely has a positive influence on outcomes in patients with severe ARDS. However, the ideal time to begin vvECMO therapy, producing the least negative side effects and the best possible positive effect on disease progression and long-term outcomes, remains unknown. By evaluating the timing of vvECMO therapy initiation, we hope to help clinicians improve care for patients with severe ARDS. Official guidelines recommend the implementation of vvECMO as rescue therapy (ie, when adequate oxygenation can no longer be sustained under lung-protective ventilation); in this context, the ELSO guidelines refer to a Horovitz index of <80, among other factors [1].

It should also be noted that there have been substantial improvements in vvECMO therapy regarding treatment and safety. For example, the incidence of bleeding complications has decreased [19]. Among other developments, recommendations on anticoagulant treatment have been changed, pointing to a more restricted use of anticoagulants on ECMO [20]. Therefore, the implementation of vvECMO earlier in the course of ARDS could be justified. Furthermore, our data might provide insight into whether, when vvECMO has been started at an earlier time point in the respective group, occurrence of bleeding complications and other adverse events are more common compared to the use of vvECMO as rescue therapy.

This trial aims to investigate an important question regarding vvECMO therapy. Even though ECMO has been used and researched for decades, the best time point for initiation of therapy is still not determined. Against the background of optimizing costly medical procedures such as ECMO therapy, from both economic and ethical aspects of society as a whole, it is important to answer the question of when to best initiate ECMO therapy.

Thorough planning involving both clinical experts and experts in clinical trials should guarantee an efficient and reliable trial process. To ensure a high enough case number and transferable results, the trial will be carried out internationally across multiple expert centers. All patients in the trial will receive standardized best medical care in ECMO centers.

One important limitation might be insufficient recruitment numbers, in which case more centers can be included in the trial. Another noteworthy consideration is the handling of patient crossover within the trial. Crossover is partially inherent to the study design, as patients randomized to the conventional treatment arm may receive ECMO as rescue therapy if predefined clinical deterioration criteria are met. This approach was chosen for ethical reasons to ensure that potentially life-saving therapy is not withheld from patients with refractory hypoxemia. To minimize uncontrolled crossover, strict criteria for ECMO initiation in the standard treatment group (rescue criteria) have been predefined and will be applied consistently across participating centers. These criteria follow established recommendations for rescue ECMO in severe ARDS and require documented failure of optimized conventional management, including lung-protective ventilation and adjunctive measures. In addition, all participating centers are experienced ECMO centers and adhere to standardized treatment protocols.

For statistical interpretation, the primary analysis will follow the intention-to-treat principle, thereby preserving the benefits of randomization. This approach allows the randomized comparison of early vs conventional ECMO strategies while simultaneously accounting for the clinically necessary use of rescue ECMO in deteriorating patients. Thus, the primary analysis refers to the comparison of 2 treatment strategies (early vs delayed ECMO) in accordance with the primary aim of our study. Secondary analysis will be performed as per protocol analysis. In the per-protocol population, participants who switched to ECMO according to the predefined criteria for the late group will be included. Patients randomized to the late ECMO group who received ECMO without fulfilling the predefined rescue criteria, or who did not switch even though these criteria were fulfilled, will be excluded from the per-protocol population. Additional secondary analyses will be performed to better understand the potential influence of crossover. These analyses will include survival analyses and exploratory time-to-ECMO analyses in the delayed treatment group.

Another potential concern that needs to be addressed is confounding by indication of vvECMO related to heterogeneity in ARDS progression. To minimize confounding in this important aspect, specific study design elements have been implemented to minimize the risk through clearly defined inclusion and exclusion criteria. As mentioned previously, reaching the criteria for severe ARDS—an oxygenation index of ≤100—is the major inclusion criterion, and study inclusion must follow within a 24-hour time frame. Additionally, we have limited study inclusion to an overall onset of ARDS within a time span of 96 hours, and mechanical ventilation must not have been initiated for >7 days. To account for confounding differences within the cohort, analysis of relevant markers of ARDS and overall disease severity—including the Horovitz index, ventilatory parameters, SOFA score, and timing of onset of chest X-ray signs—will be recorded at baseline to enable comparison between groups.

A further potentially confounding factor might be center-dependent differences in overall ARDS treatment. To lessen this effect, all participating centers are or will be expert ARDS centers with a comparable degree of expertise and resources. For ventilation strategies, the participating centers are referred to the local guidelines [9] and international recommendations [1], as mentioned previously. Naturally, the respective treatment parameters—including ventilator settings, ECMO parameters, coagulation therapy, use of prone positioning, duration of prone positioning, and additional supportive medication—will be documented daily in the eCRF and will be available for analysis.

The aim of our study is to evaluate whether initiating ECMO therapy early enables pulmonary protective ventilation at an early stage of treatment and reduces inflammation in the lungs as soon as possible, thereby reducing the side effects of ventilation. Therefore, the risk-benefit assessment is likely positive for the use of vvECMO in general and might favor an earlier initiation.

## Appendix

Multimedia Appendix 1 Overview of trial registration data.

## Abbreviations

ARDS: acute respiratory distress syndrome
CESAR: conventional ventilatory support vs ECMO for severe adult respiratory failure
DSMB: Data and Safety Monitoring Board
ECMO: extracorporeal membrane oxygenation
eCRF: electronic case report form
ELIEO: Early vs Late Initiation of veno-venous extracorporeal membrane oxygenation
ELSO: Extracorporeal Life Support Organization
EOLIA: ECMO to rescue acute lung injury in severe ARDS
FiO_2_: fraction of inspired oxygen
ICU: intensive care unit
PaO2: partial pressure of oxygen
SOFA: Sequential Organ Failure Assessment
SPIRIT: Standard Protocol Items: Recommendations for Interventional Trials
vvECMO: veno-venous extracorporeal membrane oxygenation

## Data Availability

The datasets used and/or analyzed during this study are available from the corresponding author upon reasonable request.
