# Supplementary material for: Early vs Late Initiation of Extracorporeal Membrane Oxygenation: Protocol for a Prospective, Randomized, Multicenter Study
Source: JMIR Res Protoc. 2026 Jun 17;15:e86652. doi: 10.2196/86652 (PMC13324314; doi:10.2196/86652)
Supplement: Multimedia Appendix 1 [file resprot_v15i1e86652_app1.docx]

**Multimedia Appendix 1: Overview of trial registration data**

| **Data category** | **Information** |
| --- | --- |
| Primary registry and trial identifying number | Clinicaltrials.gov  NCT04208126 |
| Date of registration in primary registry | 12/05/2019 |
| Sources of monetary or material support | Maquet Cardiopulmonary GmbH (part of Getinge Group of Companies) |
| Primary sponsor | Prof. Dr. Peter Rosenberger |
| Secondary sponsor(s) | Maquet Cardiopulmonary GmbH (part of Getinge Group of Companies) |
| Contact for public queries | peter.rosenberger@medizin.uni-tuebingen.de |
| Contact for scientific queries | peter.rosenberger@medizin.uni-tuebingen.de |
| Public title | Early Versus Late Initiation of ECMO (Extracorporal Membrane Oxygenation) Trial |
| Scientific title | Early Versus Late Initiation of ECMO (Extracorporal Membrane Oxygenation) Trial a prospective, randomized, multicenter study |
| Countries of recruitment | Germany |
| Health condition(s) or problems(s) studied | Acute Respiratory Distress Syndrome (ARDS) |
| Intervention(s) | **Group A: early ECMO initiation**  ECMO therapy will be initiated within 24 h after admission to the ICU of the ECMO center or after the inclusion criteria are met |
|  | **Group B: late ECMO initiation**  Standard treatment according to ARDS Network guidelines will be provided, with ECMO therapy as a rescue therapy only.  Rescue criteria:   - Inability to administer lung-protective ventilation - Patient is threatened by persistent or progressive hypoxemia (PaO_2_/FiO_2_ <60) or exhibits progressive acidosis (pH < 7.2) for 6 h despite measures to correct this. - Patient exhibits a blood arterial saturation of SaO_2_ <80% for >6 h despite the mandatory use of recruitment maneuvers, the inhalation of NO or prostacyclin and, if technically possible, a test of prone positioning and has no irreversible multiple organ failure |
| Key inclusion and exclusion criteria | Ages eligible for study: ≥ 18 years  Sexes eligible for study: both  Accepts healthy volunteers: no |
|  | Inclusion criteria:   1. PaO_2_/FiO_2_ ≤ 100 2. One of the following three criteria: 3. PaO_2_/FiO_2_ ≤ 100 mmHg with FiO_2_>80% > 3 h despite optimal and despite optimal recourse to adjunctive therapies 4. PaO_2_/FiO_2_ <80 mm Hg with FiO_2_ ≥80% for >3 h despite optimization of mechanical ventilation 5. pH<7.25 (PaCO≥50 mmHg) for >3 h despite optimal ventilation with a respiratory rate of up to 35 breaths/min 6. Bilateral opacities on frontal chest radiograph ≤ 7 d 7. Requirement for positive pressure ventilation via an endotracheal tube or non-invasive ventilation 8. No clinical signs of left atrial hypertension detected via echocardiography, or, if measured, a pulmonary arterial wedge pressure (PAOP) ≤ 18 mmHg 9. ≤ 96 h since the onset of ARDS   ≤ 7 d since the initiation of mechanical ventilation |
|  | Exclusion criteria:   1. Age < 18 years 2. More than 7 d since the initiation of mechanical ventilation 3. More than 96 h since meeting the inclusion criteria 4. Non-commitment from patient, surrogate or physician to full intensive care support 5. A positive pregnancy test at the time of screening 6. Cardiac failure requiring veno-arterial ECMO 7. Chronic respiratory insufficiency treated with oxygen therapy |
| Study type | Interventional |
|  | Randomized, open label |
|  | Phase: not applicable |
| Date of first enrolment | March 2025 |
| Targeted sample size | 508 |
| Recruitment status | Recruiting |
| Primary outcome(s) | Overall 90 days mortality |
| Key secondary outcome(s) | - SOFA score course - 28-d all-cause mortality - duration of mechanical ventilation - bleeding complications - ICU-related complications |
